# Sensitivity of *Aspergillus flavus* Isolates from Peanut Seeds in Georgia to Azoxystrobin, a Quinone outside Inhibitor (QoI) Fungicide

**DOI:** 10.3390/jof7040284

**Published:** 2021-04-09

**Authors:** Md Emran Ali, Mackenzie Gunn, Tammy Stackhouse, Sumyya Waliullah, Baozhu Guo, Albert Culbreath, Timothy Brenneman

**Affiliations:** 1 Department of Plant Pathology, University of Georgia, Tifton, GA 31793, USA; Mackenzie.Gunn@uga.edu (M.G.); tstackho@uga.edu (T.S.); Sumyya.Waliullah@uga.edu (S.W.); spotwilt@uga.edu (A.C.); arachis@uga.edu (T.B.); 2 USDA-ARS, Crop Genetics and Breeding Research Unit, Tifton, GA 31793, USA; baozhu.guo@usda.gov

**Keywords:** peanut, *Aspergillus flavus*, fungicide, azoxystrobin, seed infection

## Abstract

*Aspergillus flavus* infects peanuts and produces a mycotoxin called aflatoxin, a potent human carcinogen. In infected peanuts, it can also affect peanut seed quality by causing seed rot and reducing seed viability, resulting in low germination. In 2020, peanut seeds in Georgia had lower than expected germination and a high frequency of *A. flavus* contamination. A total of 76 *Aspergillus* isolates were collected from seven seed lots and their identity and in vitro reaction to QoI (quinone outside inhibitor) fungicide (azoxystrobin) were studied. The isolates were confirmed as *A. flavus* by morphological characteristics and a PCR (polymerase chain reaction)-based method using species-specific primers. In vitro, these isolates were tested for sensitivity to azoxystrobin. The mean EC_50_ values ranged from 0.12 to 297.22 μg/mL, suggesting that some isolates were resistant or tolerate to this fungicide. The sequences of cytochrome b gene from these isolates were compared and a single nucleotide mutation (36.8% isolates) was found as Cyt B G143A, which was associated with the total resistance to the QoIs. Another single mutation (15.8% isolates) was also observed as Cyt B F129L, which had been documented for QoI resistance. Therefore, a new major single mutation was detected in the *A. flavus* natural population in this study, and it might explain the cause of the bad seed quality in 2020. The high frequency of this new single nucleotide mutation exists in the natural population of *A. flavus* and results in the ineffectiveness of using azoxystrobin seed treatment. New seed treatment fungicides are needed.

## 1. Introduction

Peanuts (*Arachis hypogaea* L.) are an important source of protein and oil, and are considered to be one of the top oilseeds produced globally [1]. Global peanut production from 2010–2013 averaged over 39 metric tons per year, an increase of 136% since the 1970s [2]. The U.S. is the 4th largest producer of peanuts worldwide, following China, India, and Nigeria, producing about 5.5 million metric tons per year [1,3]. Within the U.S., the Southeast is the highest producing region, largely due to the humid subtropical-like climate with short and mild winters allowing for longer crop seasons and ideal growing conditions. While this climate is ideal for higher production and yield, it also lends itself to many limiting production factors, both biotic and abiotic [1].

Peanut yield and performance is impacted by abiotic stressors such as drought, extreme temperatures, and soil factors, as well as biotic stressors such as insects, fungal, viral, and bacterial pathogens [4]. Economically important fungal pathogens in peanuts, including stem rot, *Sclerotium rolfsii* (*Athelia rolfsii*) [5,6]; and leaf spot diseases, *Passalora arachidicola* and *Nothopassalora personata,* cause major losses and crop damage each year.

One important genus of fungal pathogens for peanuts is *Aspergillus*, a saprophytic soil fungus that contains over 200 species [6]. In peanuts, *Aspergillus* species can contaminate both pre- and post-harvest stages, resulting in spoilage, potential toxicity, and devastating economic losses [5,6]. *Aspergillus* can lead to the reduced production of peanuts through seed molding, reduced germination, and crown rot of plants in the field (*Aspergillus niger*) [7]. Some species of *Aspergillus*, including *A. flavus* and *A. parasiticus,* produce the secondary metabolite aflatoxin. Aflatoxin can cause symptoms of toxicity and death after consumption, including liver damage and carcinogenic effects in both humans and animals [6,8,9,10].

*Aspergillus flavus* is an opportunistic pathogen and can cause disease in several economically important crops, especially oil-containing crops such as maize, cottonseed, and peanuts. The fungus is present in the soil as either conidia or sclerotia, or in plant tissues as mycelia [4]. *A. flavus* has been isolated in a wide range of climates but is most commonly found in warm climate zones [5,11]. Sclerotia can survive in the soil under severe conditions for up to three years, and under favorable conditions can germinate and produce new mycelium, with population increases found under hot and drought weather conditions [5,12]. While air dispersal of conidia is possible, and associated with crops such as maize and tree nuts, soil movement and rain splash dispersal is considered to be the primary method of infection for crops such as peanut seeds and cotton [5,13,14]. Additionally, post-harvest contamination of seeds is common due to improper storage methods with excessive moisture and insect activity [15].

*A. flavus* does not always reduce yield directly, but can cause high economic losses due to contamination with aflatoxin. As aflatoxin is such a potent carcinogen, the U.S. Food and Drug Administration (FDA) limits the allowable aflatoxin concentration to 20 ppb in crops [5]. In Georgia, aflatoxin contamination on peanuts causes more than $25 million in economic losses yearly [4]. In addition to *A. flavus* being a major concern in edible peanuts, *A. flavus* is also a virulent seed and seedling pathogen, greatly reducing seed germination [15].

Currently, commercial seed treatments for *A. flavus* are limited. Dynasty PD (azoxystrobin, fludioxonil, and mefenoxam) has been the standard seed treatment in the U.S. for years [16]. Additionally, Rancona V PD (ipconazole, carboxin, and metalaxyl) has been shown to be an effective treatment, but has played a lesser role [16]. One of the active ingredients in Dynasty PD, azoxystrobin, is part of the class of quinone outside inhibitor (QoI) fungicides, first approved for use in 1966 [17]. When first introduced, QoI fungicides accounted for over 20% of the global fungicide sales within the first 10 years of their commercial offering [17]. QoI fungicides have a highly specific mode of action, inhibiting mitochondrial respiration by binding to the outer quinol-oxidation site of the cytochrome *b*c_1_ enzyme complex (complex III) [17]. Azoxystrobin is very active on *Aspergillus* spp., and, in furrow sprays, was shown to greatly reduce crown rot of peanuts caused by *A. niger* [18].

As QoI fungicides have such a specific mode of action, several important fungal crop pathogens have developed resistance, including *Alternaria alternata* [19], *Botrytis cinereal* [19], and *Blumeria graminis* [20,21]. Mutations affecting sensitivity have been identified in three regions of the mitochondrial cytochrome *b* gene (*CYTB*). Amino acid substitutions from phenylalanine to leucine at position 129 (F129L), from glycine to arginine at position 137 (G137R), and from glycine to alanine at position 143 (G143A) have been detected in *CYTB* of several phytopathogenic fungi and oomycetes that are resistant to QoIs [22]. Mutations G137R and F129L typically result in moderate resistance, which can be controlled by recommended field levels of QoI. By contrast, isolates with G143A express high resistance, with the failure of QoIs to control disease [22].

QoI resistance had not been observed in *A. flavus,* however, in early 2020, commercial peanut seed testing showed a low germination, especially of seeds treated with Dynasty PD. Further research is needed to determine the sensitivity of *A. flavus* isolated from Georgian peanut seeds to QoI fungicide, as well as to characterize *CYTB* mutations, if any, associated with the resistance phenotype. This information will be crucial to inform producers of the efficacy, or lack thereof, of QoI fungicide for *A. flavus* and to determine if other mitigation efforts are needed. The present work aimed to (a) determine in vitro sensitivity of *A. flavus* to QoI azoxystrobin, a commonly used fungicide; and (b) determine what, if any, *CYTB* mutation is present in *A. flavus* isolates collected from Georgian peanut seeds.

## 2. Materials and Methods

### 2.1. Collection of Fungal Isolates and Chemicals

In 2020, a total of 76 *Aspergillus* isolates were isolated from infected peanut seeds obtained from seven peanut seed lots in South Georgia. These isolates were cultured on potato dextrose agar (PDA) for 7 days at 25 °C to supply inoculum.

Technical-grade azoxystrobin (95% active ingredient [a.i.]; Shandong Weifang Rainbow Chemical Co., Ltd.) was used in biological activity assays. The strobilurin fungicide was dissolved in distilled water and stored at 4 °C in the dark until required. Salicylhydroxamic acid (SHAM, 99% a.i.; Acros Organics) was dissolved in methanol to prepare a stock solution (20 mg mL^−1^).

### 2.2. DNA Isolation and Molecular Identification

Fungal tissue (100 mg) was scraped into a 1.5 mL safe lock tube (Eppendorf Canada Ltd., Mississauga, ON, Canada) with steel beads for homogenization. Samples were homogenized in the FastPrep FP120 cell disruptor (Qbiogene, Carlsbad, CA, USA) for 30 s at speed 5, twice, or until there were no visible pieces of mycelia. DNA was extracted using the DNeasy plant mini kit (Qiagen, Valencia, CA, USA) according to the manufacturer’s instructions. DNA was purified using Quantum Prep PCR Kleen Spin Columns (BIO-RAD, Hercules, CA, USA). Total DNA yield and purity were estimated by measuring OD at 260 nm and 260/280 nm with a NanoDrop LITE (Thermo Scientific, Waltham, MA, USA).

Molecular identification of the isolates was done using specific primers FLA1 (5′-GTAGGGTTCCTAGCGAGCC-3′) and FLA2 (5′-GGAAAAAGATTGATTTGCGTTC-3′) targeting the internal transcribed region of the rDNA unit (ITS1-5.8S-ITS2) for *A. flavus*. Amplifications were performed using a Bio-Rad T1000 thermocycler using EconoTaq^®^ plus green 2× master mix (Lucigen, Middleton, WI, USA) following manufacturer protocol. Fungal DNA (100 ng) was used as a template for PCR reactions with the following program: 94 °C for 5 min; followed by 30 cycles at 94 °C for 30 s, 51.3 °C for 30 s, and 72 °C for 1 min; and a final extension at 72 °C for 10 min before cooling to 4 °C. The amplification products were stained with ethidium bromide after electrophoresis through a 1.5% agarose gel in 1× Tris-acetate-EDTA buffer. The purified PCR products were sequenced using Sanger sequencing (Retrogen Inc, San Diego, CA, USA) and the identity of the fragment sequences was confirmed using the Basic Local Alignment Search Tool (BLAST) analysis (https://blast.ncbi.nlm.nih.gov/Blast.cgi).

### 2.3. In Vitro Assessment of Fungicide Sensitivity

In vitro sensitivity to azoxystrobin was assessed on PDA medium using a mycelial growth inhibition assay. Salicylhydroxamic acid, dissolved in methanol, was added to autoclaved media at a final concentration of 100 µg/mL [23] when testing for azoxystrobin to inhibit the alternative respiration pathway. Autoclaved media was cooled to 50 °C and azoxystrobin was added from stocks to obtain final concentrations of 0.0, 0.1, 1.0, 10.0, and 100.0 µg/mL. Amended media was poured into 60 mm Petri plates and allowed to solidify.

To test for sensitivity, spores were harvested from 14-day-old plates by transferring dry spores with a sterile plastic loop to a 2 mL tube containing 1 mL of sterile deionized water with 0.05% Tween 20. The spore concentrations were determined with a hemacytometer and adjusted to 10^5^ spores/mL. A 5 μL-droplet was plated onto the center of an AZOXY-amended and non-amended PDA plates, which were incubated for 7 days at 25 °C before measuring the colony diameter [24]. Trials were conducted in quadruplicates and were repeated twice.

### 2.4. Amplification and Sequencing of the Cytochrome B Gene

Primer pair ASP_Lt-RSCBF1 (TATTATGAGAGATGTAAATAATG)/ASP_CtyBGSP_R2 (TCAACGTGTTTTGCACC) was used to amplify the partial cytochrome b (*CYT*B) gene fragment (~798 bp) from gDNA in *A. flavus* isolates. Polymerase chain reaction (PCR) was performed and the expected size of band obtained. PCR reaction condition were as follows: initial denaturation 94 °C for 5 m; then 40 cycles of 94 °C (15 s), 59 °C (15 s), and 72 °C (60 s) and a final extension at 72° C for 5 m in a Bio-Rad T1000 thermocycler using EconoTaq^®^ plus green 2× master mix (Lucigen, Middleton, WI, USA) following a protocol suggested by supplier. The purified PCR products were sequenced as per the abovementioned procedure. Sequences alignment was performed using Geneious software 11.1.5 to determine nucleotide and amino acid changes.

### 2.5. Resistance Phenotype Using Discriminatory Dose

Resistant phenotyping to azoxystrobin was assessed on PDA amended plates at concentrations of 2.5 μg/mL and nonamended control plates. Salicylhydroxamic acid was added to the fungicide solution, and treatments were replicated three times. Four key relative growth inhibition (RGI) categories (i) RGI for resistance, no or <15% growth inhibition; (ii) RGI for moderate resistance, 15 to 50% growth inhibition; (iii) RGI for reduced sensitivity, 51 to 75% growth inhibition; and (iv) RGI for sensitive isolates, >75% growth inhibition) were utilized to evaluate isolate sensitivity to azoxystrobin [25]. Plates were incubated for 7 days at 22 °C before measuring the radial growth in two perpendicular directions and determining fungicide sensitivity. Four plates were used for each isolate, and sensitivity tests were repeated twice.

## 3. Results

### 3.1. Fungal ID Confirmation

**Figure 1:**
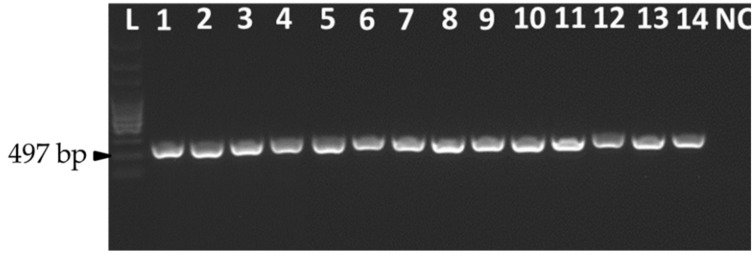
Fungal isolates confirmation targeting the ITS1-5.8S-ITS2 region of *A. flavus* isolates using the primers FLA1/FLA2. Lanes 1–14 represent isolates, while L is a 100 bp DNA molecular ladder and NC is a negative control (water only).

Initial identification of the isolates was performed based on the morphological characteristics of *A. flavus* on PDA (potato dextrose agar) plates. All the *A. flavus* isolates had a greenish colony that spread radially from the point of inoculation (Supplementary Figure S1). The identification of *A. flavus* was further confirmed using the nucleic acid-based PCR (polymerase chain reaction) method using the specific primers FLA1/FLA2 [26]. A single DNA band of 497 bp was amplified from all suspected isolates, which confirmed these isolates as *A. flavus* (Figure 1). Amplified products were then sequenced for further identification. Sequence analysis revealed that the obtained partial sequences from these isolates were 100% identical to corresponding sequences in GenBank from *A. flavus*.

### 3.2. In Vitro Assay of A. flavus Isolates to Azoxystrobin

**Figure 2:**
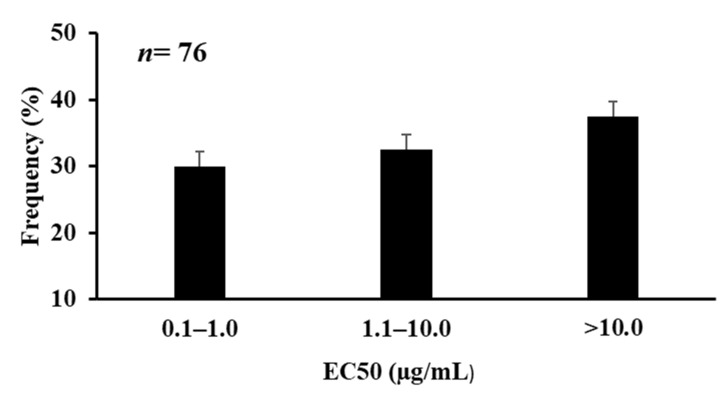
Frequency distribution of EC_50_ values (µg/mL) for azoxystrobin of *A. flavus* isolates (*n* = 76) from multiple five seed lots in Georgia.

Seventy-six isolates were used for the in vitro efficacy trial to determine the effective concentration inhibiting 50% (EC_50_) growth of *A. flavus* isolates against azoxystrobin. The mean EC_50_ value of the 76 *A. flavus* isolates to azoxystrobin was 9.07 µg/mL, and the EC_50_ ranged from 0.12–297.22 µg/mL with a variation factor of 2476.8 (Table 1). Frequencies of 76 isolates with different sensitivities to azoxystrobin were distributed in three groups in an increased pattern of EC_50_ values with an increased number of isolates (Figure 2).

The frequency distribution of EC_50_ values is shown with a normal distribution for azoxystrobin (Figure 2).

### 3.3. Detection of F129L and G143A Mutations

**Figure 3:**
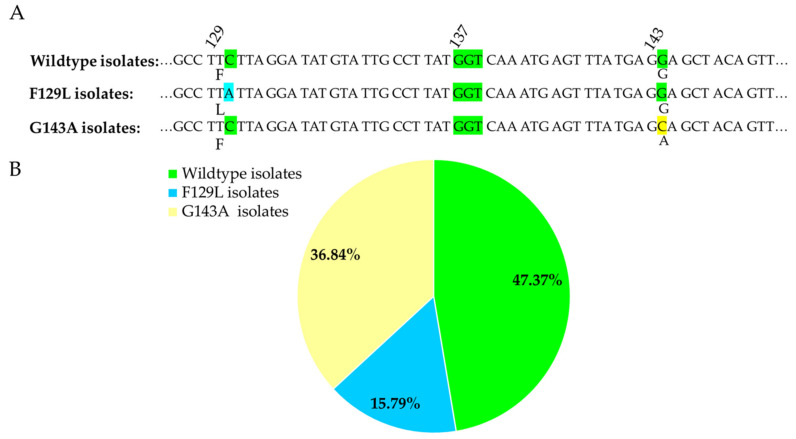
Mutation analysis of the *CYTB* gene from *A. flavus* isolates. (**A**) Sequence alignment of the *CYTB* gene from non-mutated (wild type) and mutated isolates. (**B**) A pie chart showing the percentage of isolates with or without mutation.

The *CYTB* gene of the 76 isolates was sequenced to determine if QoI fungicide target site mutations were associated with reduced sensitivity. Mutations were confirmed based on the partial sequencing of the *CYTB* gene from *A. flavus* isolates. Our sequencing analysis results showed that 36.8% of the isolates carried the G143A substitution, whereas only 15.8% of the isolates carried the F129L mutation and no isolates carried the G137R mutation (Figure 3).

### 3.4. Mutations and EC_50_ Sensitivity to Azoxystrobin

**Figure 4:**
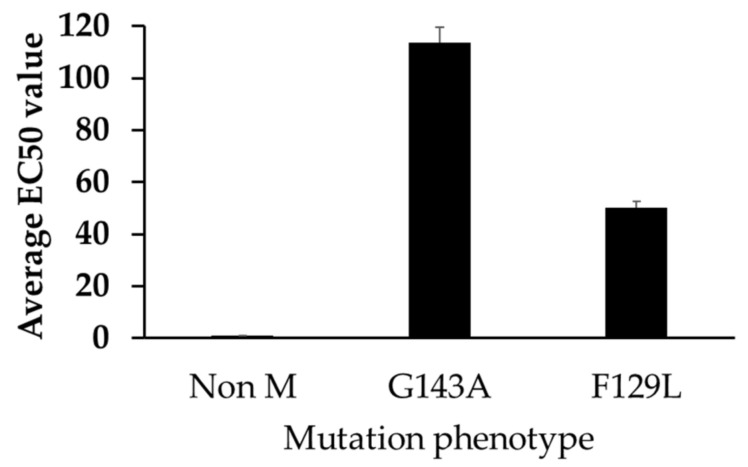
In vitro sensitivity of *A. flavus* isolates to azoxystrobin.

Our results demonstrated that the mutations detected are associated with reduced sensitivity to azoxystrobin. The mean EC_50_ values for non-mutated, or wild type, F129L and G143A isolates were 0.98, 50.30, and113.83 µg/mL, respectively, and the EC_50_ s ranged from 0.9–1.77, 1.98–182.99, and 3.16–297.22 µg/mL, respectively (Figure 4 and Table 2). The range of EC_50_ values in non-mutated isolates was narrower compared with mutated isolates, which resulted in lower VF than mutated isolates (Table 2).

### 3.5. Azoxystrobin Discriminatory Dose and Resistance Phenotype

**Figure 5:**
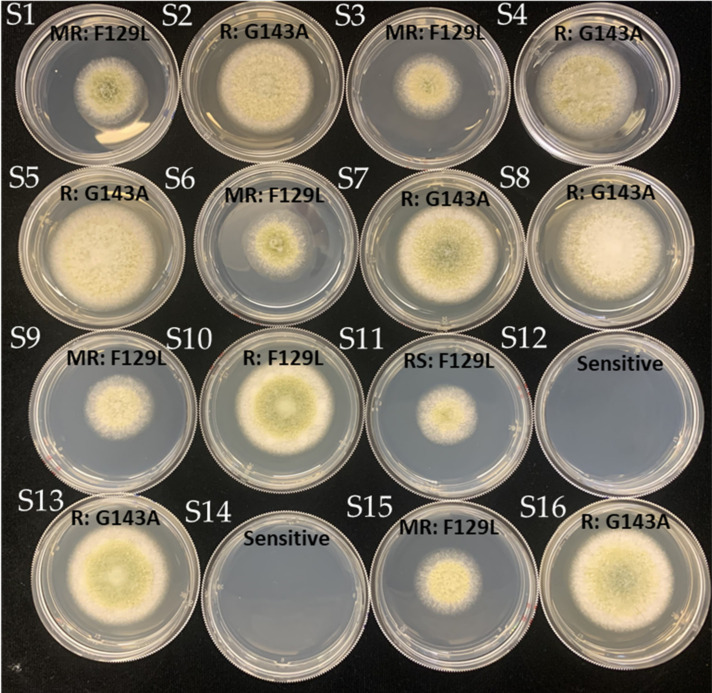
Colony morphology of *A. flavus* isolates growing on PDA amended with 2.5 ppm azoxystrobin. Isolates were categorized as either sensitive, reduced sensitivity (RS), moderately resistant (MR), or resistant (R) based on relative growth inhibition ranges.

The discriminatory dose for azoxystrobin of *A. flavus* was developed in this study based on the analysis of EC_50_ values of sensitive isolates. There is a clear distinction observed at 2.5 ppm between azoxystrobin-sensitive and -resistant isolates. All isolates were then grown at the discriminatory dose (2.5 ppm) and categorized into four groups (Figure 5). The percentage of resistant and moderately resistant isolates for G143A and F129L mutants were 86% and 50%, and 14% and 33%, respectively (Table 3). Our results demonstrated that a majority of *A. flavus* isolates were not inhibited by azoxystrobin. No G143A or F129L isolates were identified that were sensitive to azoxystrobin.

## 4. Discussion

Quinone outside inhibitor (QoI) fungicides, including strobilurins such as azoxystrobin, represent one of the most important classes of agricultural fungicides, with azoxystrobin being the largest-selling fungicides worldwide [27]. However, QoI fungicides have a high risk of resistance development in their target pathogens [27]. All QoI fungicides use a common biochemical mode of action, reducing energy production in the fungal cell through inhibition in the cytochrome *b*c_1_ complex (*CYTB)* [28]. Due to the specificity of the fungicide, just one mutation in the *CYTB* of the target fungus can result in resistance. Although QoI resistance has not been previously identified in *A. flavus* populations in Georgia, peanut seeds in 2020 showed relatively low germination, specifically in seeds treated with Dynasty PD, a commonly used fungicide containing azoxystrobin. This study provides new information on the sensitivity of *A. flavus* isolated from Georgian peanut seeds. We also characterized the *CYTB* mutation, if any, associated with the resistance phenotype. This information is crucial to inform producers of the efficacy, or lack thereof, of QoI fungicide for *A. flavus*, and to determine if other mitigation efforts are needed to minimize losses due to low germination and mycotoxin contamination.

Worldwide, resistance has been reported in an increasing number of field crop pathogens to QoI fungicides [28]. QoI resistance has now been reported in at least 20 major crop pathogens throughout Asia, Europe, and North America [27]. Our in vitro results demonstrated resistance in many of the *A. flavus* isolates collected from Georgian peanut seeds. The mean EC_50_ of the isolates collected was over 9 ppm, with a very wide range from 0.12 to 297 ppm. The wide range and variance frequency of the EC_50_ concentration of azoxystrobin throughout the isolates suggest that, overall, a proportion of the population is both moderately and highly resistant to azoxystrobin. This shift in resistance phenotype indicates that the use of azoxystrobin may have reduced efficacy in peanut seed treatment.

With resistant phenotypes found in other fungal pathogens, three mutations in the *CYTB* region (F129L, G137R, and G143A) have been commonly associated with varying efficacy of QoI fungicides [5]. Within the isolates in this study, isolates with either F129A (15.79%) or G143A (36.84%) mutations typically showed moderate to high resistance to azoxystrobin, with average EC_50_ values of 50.3 and 113.8 ppm, respectively. Compared to non-mutated isolates with an average EC_50_ of 0.98 µ g/mL, isolates with either mutation show an increased resistance to azoxystrobin. These results are similar to a preliminary report by Jordan et al. [29], where isolates of *Aspergillus* spp. section *Nigri* with the F129L mutation had reduced sensitivity to azoxystrobin compared to those with no mutation, and isolates with the G143A mutation were completely insensitive. Both of the minimum EC_50_ values found in the populations with a mutation (F129L: 1.98; G143A: 3.16 µ g/mL) were greater than the highest EC_50_ value from the non-mutated population (1.77 µ g/mL). These findings are similar to studies on other crop pathogens, such as *Botrytis cinerea* [30] and *Alternaria alternata* [31], both of which showed higher EC_50_ values in isolates with a *CYTB* mutation when compared to non-mutated isolates, indicating increased resistance to QoI fungicides.

The *A. flavus* isolates with G143A mutation had a high proportion of the population (86%) that were considered resistant to the QoI fungicide, with little to no growth inhibition at the discriminatory dose of 2.5 ppm. The remaining 14% of the population was considered moderately resistant, with some relative growth inhibition compared to the control, but not greater than 50%. None of the G143A isolates were considered to be sensitive or moderately sensitive to azoxystrobin. G143A has been shown to cause a high level of resistance in pathogens, which are consequently controlled poorly or not at all by QoIs [22]. In the model organism *Saccharomyces cerevisiae*, G143A mutation dramatically increased resistance to azoxystrobin by 4000× compared to the control [28]. Isolates with F129L mutation showed high resistance to azoxystrobin at 2.5 ppm (50%), but 33% and 17% of the isolates were considered to be moderately resistant or had reduced sensitivity to the discriminatory dose of azoxystrobin, respectively. These results are consistent with findings from other studies in which the amino acid substitution F129L has been shown to result in moderate resistance, sometimes able to be controlled by recommended field levels of QoI [5,32].

While azoxystrobin is still effective at controlling non-mutated samples, greater than 50% of the isolates collected had a mutation associated with resistance to QoI fungicides, indicating that azoxystrobin may not be a practical long-term treatment for controlling *A. flavus* infection in peanut seeds. Continual use of azoxystrobin could increase the frequency of resistant isolates, as fungicide application to an area with some resistant isolates reduces the fitness of the fungicide-sensitive individuals in the population, and allows the frequency of resistant isolates to increase faster [33]. Due to this, alternative fungicides may be required. Unfortunately, past research on QoI resistant pathogens has found that resistant pathogens with a *CYTB* mutation can also have positive cross-resistance to other QoI fungicides [32,33,34,35]. This is thought to be due to the closely related target sites and specific mode of action of QoI fungicides [36]. Consequently, other commonly used QoI fungicides such as pyraclostrobin may not be applicable for use in peanut seeds to control *A. flavus.* One study, however, did find that isolates of *B. cinerea* that were resistant to QoI fungicides showed no increased sensitivity to the Qi inhibitors (QiI) cyazofamid and antimycin-A, and QoI resistant isolates also had increased sensitivity to carboxamides and anilinopyrimidines [37]. The absence of cross-resistance between Qo and Qi inhibitors in pyraclostrobin-resistant mutants of *B. cinerea*, suggests the mutations in the configuration at Qo-site of *CYTB* may not transmit a structural change to the Qi-site, allowing resistance to binding and potential efficacy with this class of fungicide. Additionally, research on inhibitors of the aflatoxin biosynthetic pathway, such as 3-isopropylbenzaldehyde thiosemicarbazone (mHtcum), show potential as antiaflatoxigen treatments [38].

This research showed that the insensitivity to QoI fungicide azoxystrobin is present within *A. flavus* populations isolated from GA peanut seed. This phenotypic resistance is likely a result of two mutations found within the populations, F129L and G143A, conferring moderate and high resistance, respectively. These mutations have been linked to cross-resistance in other studies, indicating that QoI fungicides may not be an efficacious treatment for peanut seed to reduce losses due to *A. flavus* infection. Routine monitoring of *A. flavus* populations found in peanut seed is suggested so producers are aware of resistance phenotypes or mutations present and can make effective management decisions. Research is needed to determine the sensitivity of isolates to other seed-treating fungicides, mixtures of fungicides, or the development of new chemistries targeting a different mode of action against QoI resistant *A. flavus*. This research can be used to inform producers of the current resistance phenotypes found in Georgian peanut seeds to allow for theselection of more efficacious management practices and reduce losses due to *A. flavus* infection and resulting aflatoxin contamination.

## Figures and Tables

**Table 1:** Mean and range of effective concentration inhibiting 50% (EC_50_) growth of *A. flavus* isolates.

	*A. Flavus* Isolates to Azoxystrobin (Based on the Mycelial Growth Inhibition)
Mean EC_50_	9.07 μg/mL
Range EC_50_	0.12–297.22 μg/mL
VF *	2476.83

* VF: maximum EC50/minimum EC50.

**Table 2:** Mean and range of effective concentration inhibiting 50% (EC_50_) growth of *A. flavus* isolates.

Isolate Type	EC_50_ (μg/mL)		
	Mean	Range	VF
Non-mutated isolates	0.98	0.9–1.77	1.97
F129L isolates	50.30	1.98–182.99	92.42
G143A isolates	113.83	3.16–297.22	94.06

**Table 3:** Sensitivity of *A. flavus* isolates collected from Georgia seed lots to QoI azoxystrobin.

Sensitivity to Azoxystrobin with Four Key Relative Growth Inhibition (RGI) (%) Ranges	# of Isolates with Percentage (%) of Individual Group		
	With G143A Mutation	With F129L Mutation	Without Mutation
Resistant (0–14%)	24 (86%)	6 (50%)	0 (0%)
Moderately resistant (15–50%)	4 (14%)	4 (33%)	0 (0%)
Reduced sensitivity (51–75%)	0 (0%)	2 (17%)	2 (6%)
Sensitive (76–100%)	0 (0%)	0 (0%)	34 (94%)

## Data Availability

Not applicable.

## Supplementary Materials

The following are available online at https://www.mdpi.com/article/10.3390/jof7040284/s1, Figure S1: Colony morphology of Aspergillus flavus on peanut seed (A) and PDA plate (B).

## Abbreviations

AZOXY	azoxystrobin
PDA	potato dextrose agar
SHAM	salicylhydroxamic acid
QoI	quinone outside inhibitor
EC_50_	effective concentration inhibiting 50% growth
*CYTB*	cytochrome b
ppm	parts per million
