# Supplementary material for: Sensitivity of Aspergillus flavus Isolates from Peanut Seeds in Georgia to Azoxystrobin, a Quinone outside Inhibitor (QoI) Fungicide
Source: J Fungi (Basel). 2021 Apr 9;7(4):284. doi: 10.3390/jof7040284 (PMC8069585; doi:10.3390/jof7040284)

Supplementary file

Figure S1. Colony morphology of *Aspergillus flavus* on peanut seed (A) and PDA plate (B)

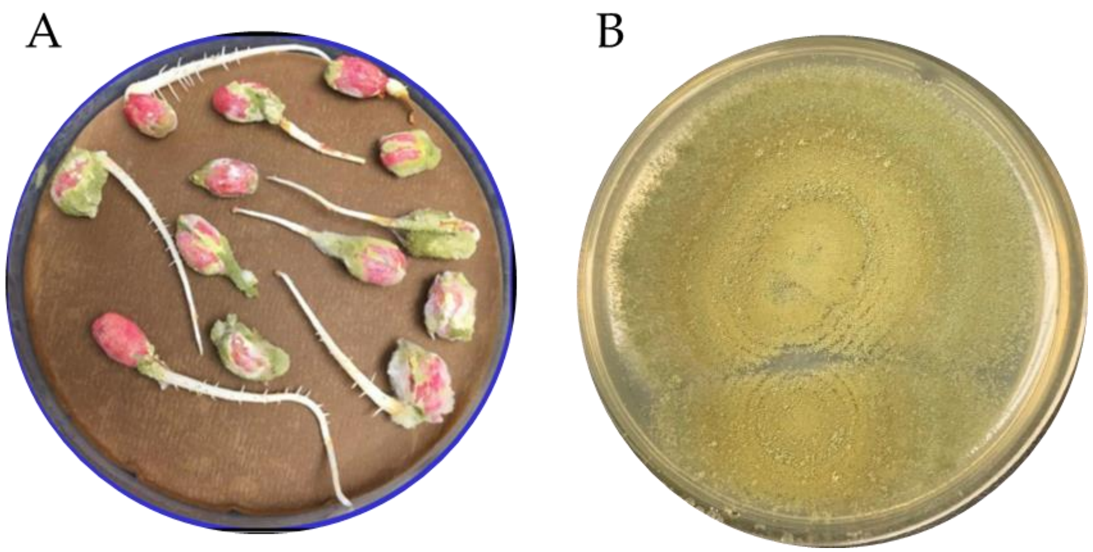

Supplement: Supplementary file 1 [file jof-07-00284-s001.pdf]
